# The Book World of Medicine and Science

**Published:** 1902-08-23

**Authors:** 


### The Book World of Medicine and Science.

Human Embryology and Morphology. By Arthur Keith, M.D. (Aberdn.), F.R.C.S. (Eng). (London: Edward Arnold. 1902. Price 12s. 6d., net.)

In dealing with such a subject as is here treated of, the author is, of necessity, even from the outset, met by a great difficulty, that, namely, of determining how far he will go, and at what limit he will draw rein in his excursion into a field, the range of which is so immense as is that covered by embryology and comparative anatomy. Perhaps this difficulty may explain the fact that in the book before us, all parts and all branches of the subject are not treated with quite the same degree of minuteness. The rule which the author has laid down for his guidance in the matter is, however, a good one. He has endeavoured to deal with each portion of his subject to an extent determined by its practical importance to the medical student. The course of demonstrations, of which this book is the substance, was given at the school connected with a great hospital, and, as the preface tells us, "clinical utility was the criterion employed" in determining the scope of the work. From end to end the book is descriptive. Without a word of introduction the author plunges into the subject of the "development of the face," while, equally without a word of moral, the last chapter ends with the last words of a description of the embryology of the lower limbs. From beginning to end the whole volume is full of careful, well sustained, and, so far as the subject allows, of interesting description of what happens to the different parts of the human body during their embryonic growth, and of comparisons between the process in man and in other animals. The first thing that strikes one in turning over the pages of this work is the clearness and definiteness of the illustrations, the freedom with which they have been introduced, and the care with which each is made to render plain some point in the text. The next is the exactitude of the descriptions given of the very curious and abstruse matters dealt with. It is an admirable book, and for the student who wishes, as every student must wish, although he may not always attain to his desire, to know how all those things grew which he sees before him in his dissections, it should prove an endless source of instruction. It is not, however, an amusing book; and, read by itself, a little goes a long way. To get the full benefit of it one's brain must be fully charged with anatomical lore; then every page is explanatory of the unexplained.

Tendencies to Consumption, and How to Counteract Them. By T. D. E. Mortimer, M.B., F.R.C.S. (London: The Scientific Press, Limited. 1901. Pp. 135, cloth boards. Price 2s. 6d.)

Dr. Mortimer has produced a really excellent little book: very much to the point, and strikingly free from the pseudo-science and verbiage now so much *en evidence*. "Tendencies to Consumption" is well written, and does not lack the imperative tone so necessary to arrest public attention. In the first part the nature of consumption is clearly explained, and a due sense of proportion is displayed in dealing with the various predisposing and auxiliary factors in its causation. The second part is a forcible exposition of how consumption may be prevented, and is far from being a mere *rechauffé* of text-books of hygiene. The chapter on "Exercise and Recreation" and that on "Infancy, Childhood, and Adolescence" strike us as peculiarly valuable, and the truths which they embody are expressed with an incisiveness which is at times epigrammatic. The concluding sentences of Dr. Mortimer's book are at the present time particularly apposite and deserve quotation:—"Sunshine and pure air, nourishing but unstimulating food, moderate exercise, early hours, abstinence from excessive toil . . . . and from all kinds of exhausting indulgences":—These are the topics on which Sir Thomas Watson insisted half a century ago, and a physician of the present day can "only admire his wisdom and emphatically repeat his advice." Legislation is urgently needed to regulate the purity of milk and meat, and also, as regards a large bulk of the population, the supply of fresh air and of means by which cleanliness may be easy instead of difficult. It might be even better for philanthropists to try and arouse the Legislature in regard to these questions than to expend money and energy on behalf of those already affected."

Some Results of the Sanatorium Treatment of Phthisis. By Chowry Muthu, M.D. (London: T. Ball, Sons, and Daniellson. 1901. Pp. 43, paper.)

This pamphlet is a reprint, with appendices, of a paper presented by Dr. Muthu to the British Medical Association at Ipswich last year. It gives a brief account of the system carried out by the author at the Sanatorium of which he is the directing physician. It cannot be said that there is anything very original in the detail of Dr. Muthu's system, and we note that the Sanatorium appears to be not a specially planned building, but an "adapted" mansion. Coming to that portion of the pamphlet in which most interest is excited by the title, we learn that of 22 patients treated during the first 11 months, five entirely recovered. Dr. Muthu means, when he says "entirely recovered," that "no physical signs were present" and no tubercle bacilli found in the sputum when the patients left. We should welcome some ampler clinical detail than these bald statements as to the "absence of physical signs." Dr. Muthu states, however, that certain cases are not very favourable to open-air treatment, and amongst these are:—Chronic cases that have run a mild course for a long duration, cases with feeble stomach and nausea, cases with weak cardiac action, cases with bronchial catarrh and incessant cough, and cases below 20 years of age. Dr. Muthu, with commendable candour, further points out that exposure to strong winds, especially when easterly, has a most depressing and irritating effect, and that many patients who fail to benefit by the new treatment do benefit by change of climate.

Matriculation Directory, No. 32. 1902. (Burlington House, Cambridge. Pp. 120. Price 1s.)

This volume is one of the University Tutorial Series, issued under the auspices of the University Correspondence College. In spite of its rather pretentious title we find it to consist of the Calendar of the University Correspondence College, and the questions set at the recent "London Matric." To these questions there is appended a set of "solutions" provided by the tutors of the University Correspondence College. There is also an abstract of the old and the new regulations for the Matriculation Examination.

